# Pendulum-Based River Current Energy Converter for Hydrometric Monitoring Systems

**DOI:** 10.3390/s22114246

**Published:** 2022-06-02

**Authors:** Slim Naifar, Felix Grimmeisen, Christian Viehweger, Zheng Hu, Arthur Bauer, Peter Hörschelmann, Olfa Kanoun

**Affiliations:** 1 Chair for Measurement and Sensor Technology, Technische Universität Chemnitz, Reichenhainerstraße 70, 09126 Chemnitz, Germany; christian.viehweger@etit.tu-chemnitz.de (C.V.); zheng.hu@etit.tu-chemnitz.de (Z.H.); olfa.kanoun@etit.tu-chemnitz.de (O.K.); 2 SEBA Hydrometrie GmbH & Co. KG (SEBA), Gewerbestraße 61a, 87600 Kaufbeuren, Germany; grimmeisen@seba.de (F.G.); a.bauer@seba.de (A.B.); 3 Institute of Applied Geosciences (AGW), Karsruhe Institute of Technology (KIT), Kaiserstraße 12, 76131 Karlsruhe, Germany; 4 JuB-Creative Product GmbH, Industriestraße 12, 99846 Seebach, Germany; p.hoerschelmann@jub-cp.de

**Keywords:** energy harvesting, kinetic converter, hydrometric monitoring system, autonomous systems, water flow

## Abstract

Energy harvesting from flowing water is important for supplying hydrometric monitoring systems. Nevertheless, it is challenging due to the chaotic water flow in only one main direction and the relatively weak energy profile. In this paper, a novel energy harvester has been proposed, designed, and validated. The converter consists of a pendulum, a gearbox, two overrunning clutches, a spiral spring, and a generator. By coupling the kinetic energy via an oscillating mass equipped with a magnetic spring, it is possible to accommodate the power supply, electronics, and sensors with data transmission in a completely closed, encapsulated, stable housing without an interface to the outside. In addition, an energy management circuit and a battery charging circuit were developed that could be housed in the sealed enclosure. The pendulum transducer prototype was tested with the developed online hydrometric measurement station, which consists of a multi-channel data logger with a cellular modem and a tipping bucket rain gauge sensor. The overall system was successfully validated by experimental studies in a river.

## 1. Introduction

With the increase in demand for distributed sensor networks, great focus has been devoted to harvesting environmental energy in order to replace batteries and extend the sensor nodes’ lifetime [1,2,3,4] in wireless sensor networks. Considering the cost and the environmental issues, ambient energy provides key benefits to be considered as one of the major sources of energy in the future [5,6]. Furthermore, environmental energy harvesters can increase the self-powered micro device’s adaptability. So far, numerous harvesting devices have been designed to convert environmental energy like solar [7] or thermal power [8], vibration and kinetic energy [9] into electrical power. Specifically, fluid kinetic energy is considered one of the most easily reachable and renewable energy sources in the environment [10]. In this context, hydrokinetic energy of flowing water in rivers presents a potential source of energy for powering wireless sensor systems such as hydrometric monitoring systems [11].

Fluid kinetic energy is mostly harvested by traditional hydro-turbine structures, which are able to harness the energy of moving or falling water [12]. Hydroelectric systems are mainly used at large scales for generating electrical power [13,14]. Water has also received great attention for small-scale energy harvesting to produce electrical power [15]. For example, in [16], a commercial hydrogenerator developed and patented by Vulcano is reported. The harvester was installed in an irrigation pipe and is able to generate almost constant output power in the range of 18 mW.

An alternative solution is to use fluid dynamics structures to induce a structure vibration, through which the fluid kinetic energy can be converted into vibration energy, and then, use a different method, including piezoelectricity and electromagnetism, to finally convert vibration energy into electrical energy. As a result, an eel-like hydraulic power harvester has been designed to convert ocean current into electrical power [17]. Piezoelectric eel energy harvesters are composite devices made of piezoelectric polymers deposited on structural supports. The eels are generally placed in river flows behind a bluff body used to generate vortexes [18].

Shan et al. presented a piezoelectric energy harvester (PEH) made from macro fiber composites (MFC) that is immersed in water vortexes caused by cylinders upstream [19]. The PEH consists of an MFC (M8514-P2) beam and a polyvinyl chloride (PVC) layer. The results show that the output power increases with the increase of the flow velocity, which varied from 0.05 m/s to 0.5 m/s. A maximum power output of 1.32 μW corresponding to a power density 1.1 mW/m${}^{2}$ was obtained at the water velocity of 0.5 m/s and a cylinder diameter of 30 mm. In [20], authors reported on an upright vortex-induced piezoelectric energy harvester fabricated by a piezoelectric cantilever beam and a cylinder to harvest energy from water flow. The results show that the maximum output power of the harvester is 84.49 μW with an energy density of 60.35 mW/m${}^{2}$ at a velocity of 0.35 m/s. In [10], harvest water flow induced vibration with a tandem arrangement of two piezoelectric energy harvesters (PEHs) in the direction of flowing water. Results indicate that the maximum output power of the downstream piezoelectric energy harvester (DPEH) reaches 371.7 μW, which is approximately 2.56 times of that of the upper piezoelectric energy harvester (UPEH), at a specific spacing between the upstream and the downstream piezoelectric energy harvesters.

However, most of reported water flow harvesters were investigated under laboratory conditions. Further, all of them have an active element in contact with the flowing water, which may significantly affect the reliability of such solutions when considering real implementation.

This paper addresses this issue by eliminating the contact between the flowing water and the converter while maintaining its ability to harvest a sufficient amount of energy to power the wireless sensor system. This paper presents an energy-autonomous measuring system that can use the naturally available kinetic energy in a hydrometric measuring station’s environment, thereby enabling low-maintenance measurements and modern remote data transmission. Contrary to traditional water flow harvesters, the developed converter is able to power the measuring station in a completely closed, encapsulated, stable housing without an interface to the outside. This paper is structured as follows: As part of Section 2, the design configuration including river flow analysis, converter working principle and an equivalent physical model are presented. The developed energy management and battery charging circuits are described in Section 3. Additionally, the hydrometric monitoring system and its current consumption are assessed. The results of laboratory characterization and field measurements conducted in a river are discussed in Section 4.

## 2. Device Configuration

**Figure 1:**
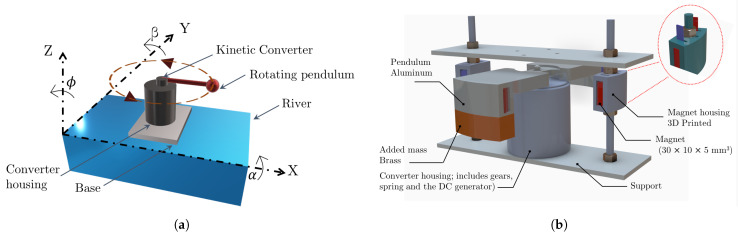
(**a**) Schematic of a pendulum-based kinetic converter on the river, (**b**) improved pendulum design for river current energy harvesting.

The kinetic converter consists of a rotating pendulum, which is connected to a bi-directional mechanism, a spiral torsion spring, gears, and a DC generator having a working principle similar to that of kinetic wristwatches. The converter is placed close to the center of gravity of a rectangular base, which is floating on the surface of the river and attached from one of its corners to a fixed frame. Figure 1a shows the schematic of a rotating pendulum along with the coordinate axes where α, β, and ϕ represents respectively the roll, pitch, and yaw angles. In the configuration under consideration, the pendulum, which is presented by a weight suspended from a pivot, is at horizontal position. Under roll and pitch oscillations, the pendulum is able to rotate in the XY plane.

On the other side, as power consumption of hydrological measurement systems significantly exceeds that in the case of wristwatches, a significantly heavier weight has to be associated with the kinetic converter to be able to harvest a valuable amount of energy from river water flow. The challenge thereby is, as the river water flow excites the kinetic harvester, the heavy pendulum must be quickly stabilized in a static position parallel to the direction of the applied excitation and will then require a large excitation in a different direction to turn again.

### 2.1. River Flow Analysis

It is particularly important to consider the position of the converter in the river. Random placement of the converter directly in the river will require continuous adjustment of its position based on different parameters, such as the river flow rate. Ideally, the kinetic converter should be located where the flow oscillates in time. In order to determine potential locations of the converter, we propose to analyse river water flow analysis. River flows can be considered as quasi-one-dimensional channels with variable and rough cross-sections. Water in the river past a fixed obstacle is of particular interest. The Reynolds number largely determines the qualitative behavior of fluid flow over an obstacle. Natural flows are usually turbulent due to the large Reynolds number $R_{e}$, which can be determined for a river by the following formula:

(1) $$R_{e}=\frac{Q}{\mu l}$$

where *Q* is the volume flow rate, *l* is the length of the river, and μ is the kinematic viscosity.

**Figure 2:**
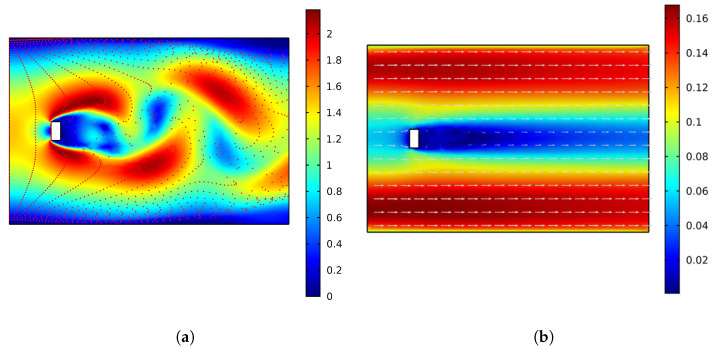
Comparative finite element analysis of laminar and turbulent flows: (**a**) model built with laminar flow interface where $R_{e}$≈ 50; velocity magnitude in m/s—red points present particle tracing with mass; drag-driven particle movement; (**b**) model built with turbulent flow k−ϵ interface where $R_{e}$≈ 5 × 10${}^{5}$; velocity field in m/s—arrows represent the fluid flow vectors.

Nevertheless, a comparative finite element analysis study of flows passing through an obstacle for different $R_{e}$ values is presented using Computational Fluid Dynamics (CFD) module of Comsol Multiphysics version 5.4. The study is performed under similar conditions in terms of the size of the canal and that of the obstacle. Specifically, the study is realized in a 6 m length and 4 m width canal with a rectangular obstacle having width and length of 0.2 and 0.4 m (see Figure 2a). Both laminar flow and k−ϵ interfaces in Comsol were employed. For low Reynolds $R_{e}$ values (below 100), the flow is steady. A 2D model is developed to simulate the flow at relatively low $R_{e}$ values using laminar flow interface, and results are presented in Figure 2a. On the left boundary, a constant inlet velocity is ramped up over time with a mean inflow velocity of 0.1 m/s. In addition, a constant outlet pressure is defined on the right boundary.

In contrast, a 3D model is developed to simulate the flow at higher $R_{e}$ values (3 × 10${}^{5}$ < $R_{e}$ < 3.5 × 10${}^{6}$) in a canal of a 6 m length, 4 m width, and 1 m height with water flowing through it at a velocity of 0.1 m/s. In this case, the underlying Navier–Stokes equations for flows are solved with a RANS turbulence model using the Comsol module “Turbulent flow, k−ϵ interface” (see Figure 2b).

Results are depicted in Figure 2a,b for the models built using the laminar and the turbulent flow interfaces, respectively. Figure 2a shows alternating vortex street forms where the vortices and streamlines are laminar (Kármán vortex street), where the maximum velocity magnitude is approximately 2 m/s. In Figure 2b, there are no clear vortex streets to be observed. The flow is laminar with an unsteady and turbulent wake where the maximum velocity magnitude is approximately 0.16 m/s. These results indicate that harvesting energy from a flow using a standard pendulum structure is difficult, as the oscillation in time in the flow depends on several parameters, including the velocity of inflow and the kinematic viscosity values, which are additionally affected by the temperature.

This paper presents a new pendulum design based on restoring forces as a method of harvesting flow energy even in the absence of vortex streets. Additionally, the proposed solution can increase the converter’s sensitivity to unidirectional and small excitations. The magnetic pendulum design is based on a heavy weight having a specific geometry and is made of non-magnetic material with drag wings. A hole is drilled in one wing, and an additional mass is added to the end of the second wing to create an unbalanced structure. Four rectangular magnets are placed near the ends of the lateral wings. Four additional magnets are attached to two outer magnet housings so that a repulsive force is created between the magnets. The magnet holder is attached to the kinetic transducer through the housing, as shown in Figure 1b.

### 2.2. Working Principle of the Kinetic Converter

**Figure 3:**
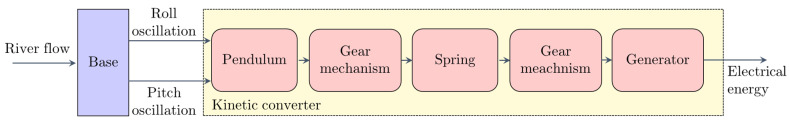
Block diagram representing the river flow kinetic energy converter.

Figure 3 shows the energy conversion of the input river flow into electricity through the designed kinetic converter. The converter is formed of several mechanical components to store the input kinetic energy at a determined level before transmitting it to a DC generator. It is mainly composed of a pendulum, gears including a bi-directional mechanism, a spiral torsion spring with a magnetic casing, and a DC generator.

Based on the bi-directional mechanism, the rotating pendulum is able to transmit its rotary motion via its gear wheel to one drive gear wheel of each of two directionally actuated couplings. One coupling transmits the torque to its output shaft when the direction of rotation is clockwise, the other transmits the torque to its associated output shaft when the direction of rotation is counterclockwise. If the directional clutches are each driven in the opposite direction of rotation—and this also applies when rotary motion is initiated from the output side—they run freely, i.e., they do not transmit torque in these opposite directions, neither from the input side to the output side nor vice versa from the output side to the input side. The permanently excited DC generator builds up a counter-torque through its own cogging torque, which keeps the output wheel of the spring mechanism in the idle state until a specific torque is exceeded. Only when the spring tension exceeds a specific value as a result of the winding and there is thus a slightly higher torque on the spring accumulator output wheel compared to the generator detent torque acting directly or indirectly is the movement to drive the generator is triggered. The harvested energy is stored in the spring, which releases when reaching a predefined holding torque, which is controlled through the magnetic casing acting on the spiral spring. This structure makes the obtained output from the DC generator regular with a fixed amplitude.

### 2.3. Physical Modelling

In order to replicate the behaviour of the kinetic converter, an equivalent physical model of the kinetic converter is developed in MATLAB version R2019a, Simulink version 9.3, and Simscape version 4.6. Both Simulink and Simscape library blocks are used to reproduce the behavior of the converter.

**Figure 4:**
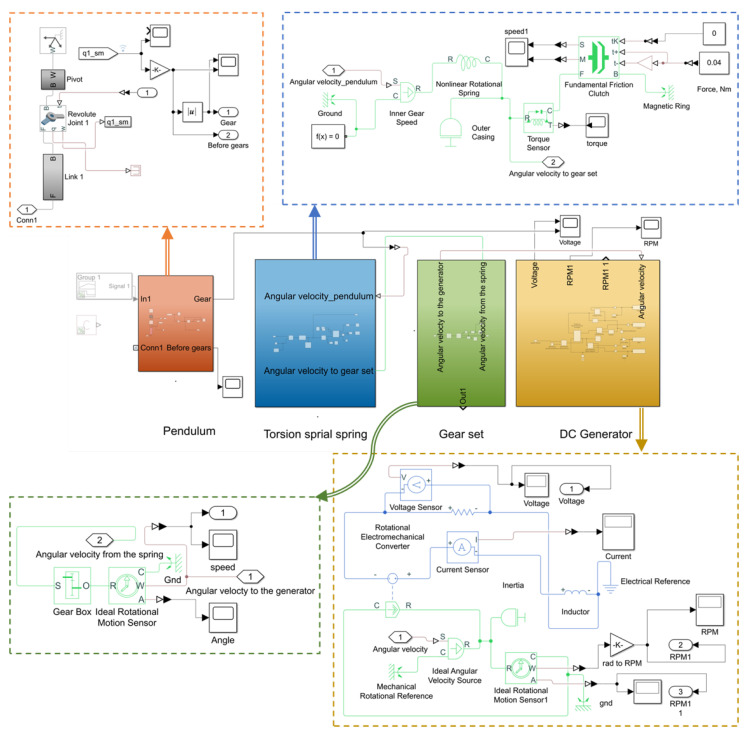
Developed Matlab/Simulink model of the kinetic energy converter.

The model is composed of four main blocks, which are presented in Figure 4 as follows:

(a) Pendulum:
  The input of the pendulum model is the acceleration in m/s${}^{2}$; the output is the angular velocity in rad/s. The pendulum design can be developed directly in the model or imported as a CAD file. In the model under consideration, the pendulum is represented by a rigid body formed by a string and a bob block. The bi-directional mechanism is included in the pendulum model. A graphical representation of the resulting pendulum rotation is included in order to count the number of pendulum turns as a function of the applied acceleration. In this case, the number of pendulum turns refers to a 360-degree rotation of the pendulum without considering the magnetic solution presented in Figure 1b.
(b) Torsion spiral spring:
  A nonlinear spiral torsion spring is configured by setting the parameters of the spring constant. For the nonlinear torsion spring, the deformation vector and the torque vector are configured depending on the properties of the spring. The selected spring is made of rectangular section austenitic stainless steel, number 1.4310 grade X10CrNi18-8, having 0.2 mm thickness and 10 mm width. The inner and outer diameters of the wound spring are, respectively, 20 mm and 48 mm. To reproduce the behavior of the magnetic force and the kinetic friction between the spring and the magnet housing, a fundamental friction clutch coupling is employed. Inputs and outputs of this block are coupled with kinetic and static force values. The clutch remains locked unless the torque transmitted through the clutch falls outside the static friction limits, which are determined as a function of the DC generator holding torque, which in turn depends on the coupled resistance load value. As soon as the spring winding torque overcomes the static friction of the clutch, it begins to unwind.
(c) Gear set:
  The model of the engaged gear is based on the transmission ratio. In the considered model, it is equal to 0.0211.
(c) DC generator:
  A DC machine block is selected to model the DC generator. Several parameters such as the coil resistance, inductance, and torque constant are included to build the model of the DC generator. In addition, the load characteristics of the DC generator and their impacts on other parameters were examined. The DC generator used in this work is a commercially available DC motor model FF-130 SH-11340 having an operating range between 3 to 12 V. At no load, the motor has a speed of 6100 r/min and a current of approximately 0.033 A.

For the simulation model, it is worth noting that no mechanical losses due to air resistance and friction were considered. Table 1 presents different parameters that were used in the developed physical model.

The complete system is studied under pulse excitations acting on the pendulum. By defining the generator holding torque, we can study the magnitude of the generated pulses in addition to the pulses repetition frequency. Once the device parameters, such as the spiral spring properties, the magnetic casing, the DC generators, and the gear ratio, are fixed, the only parameter that can affect the holding torque is the load resistance across the generator terminals. Thus, by varying the holding torque as a function of the load resistance, the amount of energy stored in the spring before release changes, which needs to be analyzed to find a balance between converter efficiency and the requirements of the energy management and storage circuits, which will be presented in the next section. To that end, we propose to analyze the converter generated pulses as a function of several load resistances while calculating the required number of pendulum turns required to spring release.

**Figure 5:**
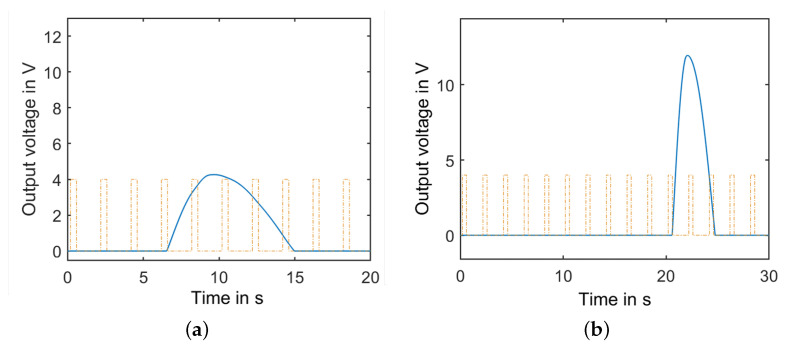

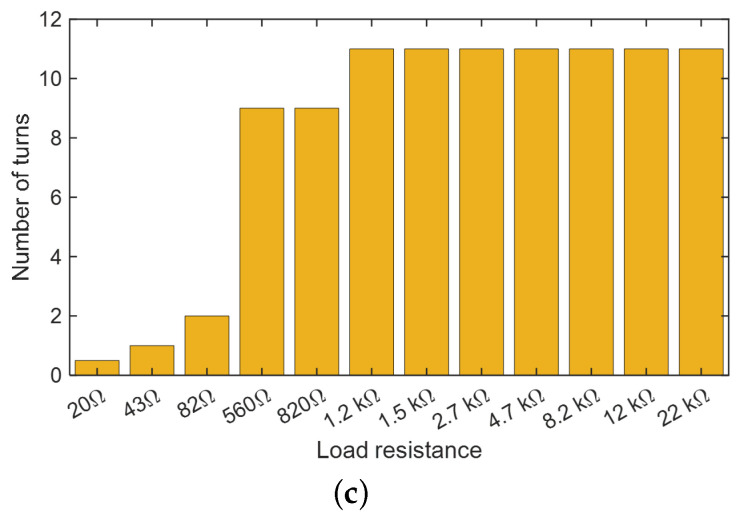
Generated pulses from the DC generator for different load resistances: (**a**) 82 Ω, (**b**) 4.7 KΩ. Rectangular signal presents the pendulum turns. (**c**) Pendulum number of turns required to release as function of several load resistances.

For instance, the simulated generator output voltage under 82 Ω and 4.7 kΩ load resistances is shown in Figure 5. For the case of 82 Ω load resistance, a maximum voltage output of approximately 4 V is obtained. The pulse is observed after complete turns of the pendulum. For the case of 4.7 kΩ load resistance, the output voltage has a high amplitude level of approximately 12 V, and it requires 11 pendulum turns. Figure 5c shows the pendulum number of turns required to release as function of several load resistances from 20 Ω to 22 kΩ. In the next section, the power consumption of the hydrometric monitoring system is investigated, which will allow us to select the suitable converter parameter.

## 3. Hydrometric Monitoring System

**Figure 6:**
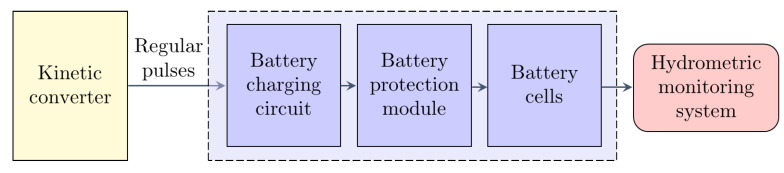
Block diagram of the autonomous hydrometric monitoring system.

A block diagram of the autonomous monitoring system is shown in Figure 6, which can be powered by the kinetic converter. In the following section, the hydrometric monitoring system is presented with a detailed analysis of its daily current consumption based on experimental investigations.

### 3.1. System Design and Power Consumption

**Figure 7:**
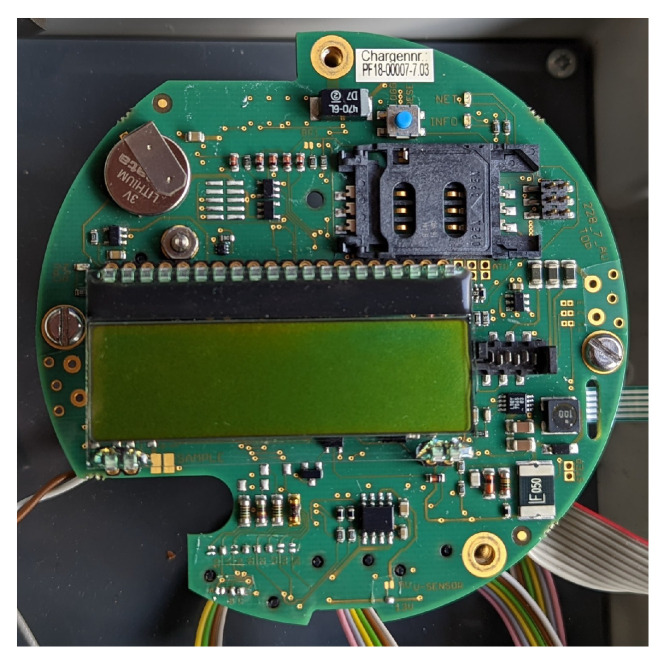
Electronic platform UniLogCom.

The used hydrometric monitoring system enables data transmission based on 3G and 4G standards. For this purpose, an electronic platform of the data logger device with new modem hardware and firmware was developed (SEBA types UniLogCom 4G and SlimLogCom 4G). The development work on the hardware and firmware for integrating the 4G modem finally enabled decisive and increased flexibility with even lower power consumption. Figure 7 shows the developed electronic platform UniLogCom with integrated 4G modem.

In order to know the power consumption of the electronic platform, extensive power consumption tests were undertaken. For this purpose, the UniLogCom data logger was connected to the tipping bucket precipitation sensor (SEBA type RG50).

The power consumption test series was performed using the UniLogCom according to the following procedure with three measurements each:

- Rain pulse measurement (RG50);
- Connection setup with RS232 interface;
- FTP push remote data transfer;
- 8-min data retrieval from modem.

**Figure 8:**
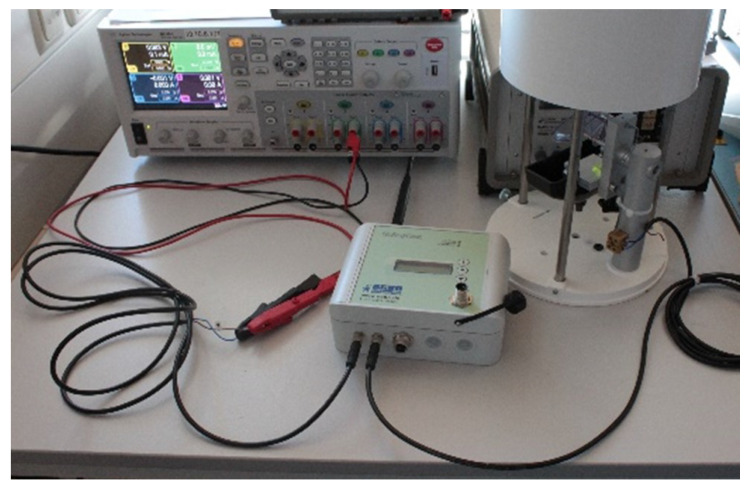
Test setup for the accurate recording of power consumption.

The UniLogCom is able to operate with a voltage level between 6 and 15 V. To that end, we performed the current consumption investigations with 6 V and 12 V supply per test series, and the modem functions were activated or deactivated. Figure 8 shows the setup used for the experimental power consumption investigations.

A precipitation sensor RG50 tipping bucket was used in the power consumption tests. The RG50 is a highly accurate precipitation gauge with pulse output for data collector systems. It has a plastic rocker with ball bearings on one side and a bubble level and adjustment screw. The precipitation gauge can optionally be equipped with a heater so that it can also be used at temperatures below freezing. The RG50 precipitation gauge is used exclusively for recording precipitation quantities in the form of pulse discharges.

**Figure 9:**
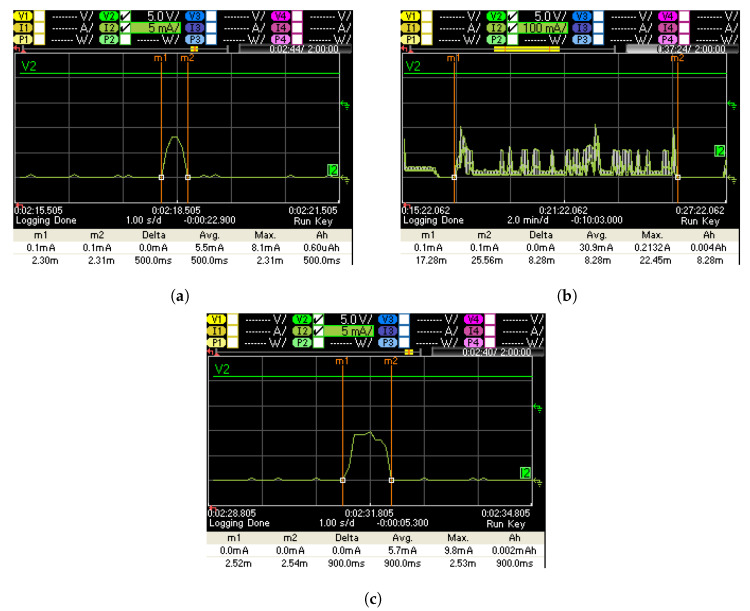
(**a**) Current consumption rain pulse—measurement 1 at 6 V, (**b**) current consumption minutes time slot + data retrieval—measurement 1 at 6 V, (**c**) current consumption logging interval 00:00 h—measurement 1 at 6 V.

(a) Current consumption measurement rain pulse (RG50):
  The UniLogCom was supplied with 6 V/12 V and the modem functions were deactivated. The rain rocker was pressed once to record the current pulse. Measured signal at 6 V is shown in Figure 9a.
(b) Power consumption measurement 8 minutes modem data retrieval:
  The UniLogCom was supplied with 6 V/12 V and the time slot for data retrieval was activated. During the active 8 minute time slot, a manual data retrieval was triggered. (CH01, CH2, and CH3 data size < 1 kB). Measured signal at 6V is shown in Figure 9b.
(c) Power consumption test logging interval:
  The UniLogCom was supplied with 6 V/12 V and the modem functions were deactivated. The logging interval, which is to be expected daily at 00:00, was mapped below. During the measurement CH03—rain sum and CH32—supply are recorded. Measured signal at 6 V is shown in Figure 9c.

Results of the daily current consumption of the UniLogCom with RG50 sensor with 6 V and 12 V supplies are shown in Table 2 and Table 3, respectively.

### 3.2. Energy Management and Battery Charging Circuits

In order to link the energy generation and the measurement task in a defined way, an intelligent energy management is of central importance. This part is particularly challenging due to the influence of the solution on the behavior of the transducer and the challenge of selecting a storage element suitable for the intended application in terms of intermittent energy extracted from the ambient source and intermittent use of the stored energy depending on the hydrological measurement system. Based on the current consumption investigation, a storage element consisting of two series-connected Li-ion batteries of 3.7 V each is used to power the hydrological measurement system. To protect the battery from being overcharged, a commercial voltage protection circuit based on HY2120 is inserted between the interfaces of the battery charger to the batteries [21]. The state of the battery group can be captured by this IC. Together with power MOSFET with a parallel body diode, this IC can decide the current flowing orientation through its over-charging and discharging controlling logic: When the overcharging state is triggered, the power generated by the battery charger cannot be injected into the battery group; when the dis-overcharging state is trigged, the battery will not deliver its power to the measurement device. Table 4 shows an overview of the main components used for the realization of the energy management and storage element.

**Figure 10:**
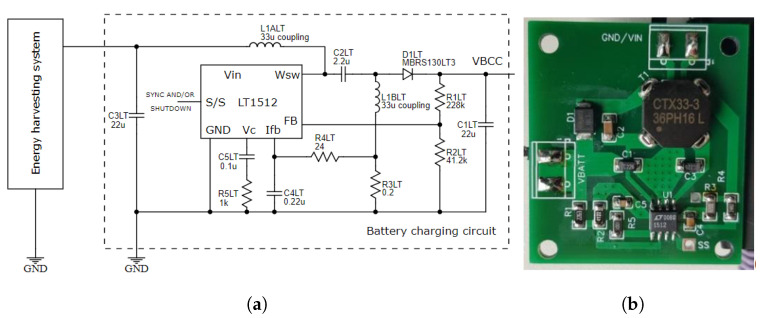
Constant current/constant voltage battery charger: (**a**) schematic of the circuit, (**b**) designed PCB.

The charging circuit is shown in Figure 10, which is mainly based on LT1512. The LT1512 is an IC battery charging chip specifically optimized to use the single-ended primary inductance converter (SEPIC) topology. This special topology has unique advantages for battery charging: it operates at input voltages above, equal to, or below the battery voltage; it has no off-state battery discharge path, and eliminates the snubber losses of flyback designs. This IC also has a current measurement point that is ground referenced and does not need to be connected directly to the battery.

## 4. Experimental Validation

### 4.1. Laboratory Characterization

**Figure 11:**
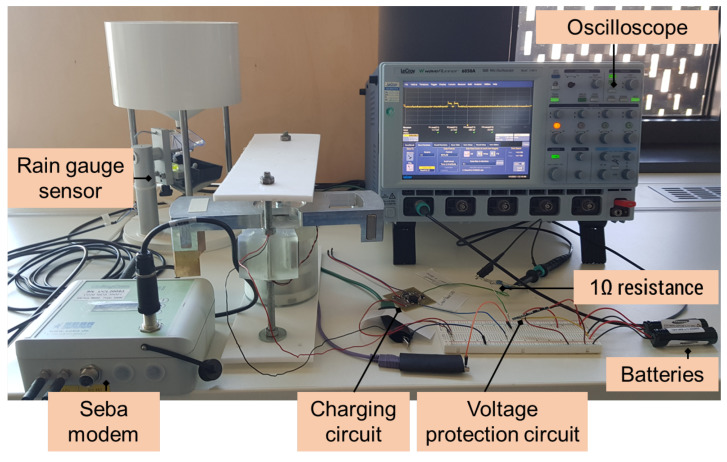
Experimental setup for laboratory characterization of the energy management and storage circuits.

**Figure 12:**
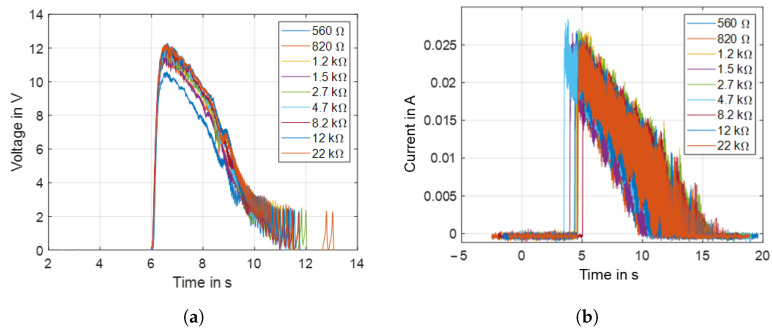
(**a**) Open circuit voltage output, (**b**) charging current to the batteries.

The characterization and evaluation of the energy management circuit is performed through laboratory tests with the developed converter and hydrometric measurement system, including a rain gauge sensor, as shown in Figure 11. Because the load resistance connected to the converter output can change the characteristics of the voltage output in terms of pulse amplitude and width, the output response under several load resistances was first investigated. Figure 12a,b, respectively, show the open-circuit voltage output of the converter and the charging current to the batteries for load resistances from 560 Ω to 22 kΩ. Results are shown in Figure 12. For load resistances below 560 Ω, the open-circuit output voltage of the converter is below 2 V, which is below the minimum voltage level required for battery charging circuit operation.

To determine the optimum load resistance, both the efficiency of the kinetic converter and the efficiency of the charging circuit must be considered. For this purpose, the generated power per trip was evaluated under different load resistances and the generated power per pendulum revolutions was determined (see Table 5). The results show that the optimum load resistance is approximately 4.7 kΩ, with the generated power in the generator being released at 0.0303 mAh at 8.4 V, which requires 11 complete pendulum turns.

### 4.2. Field Measurement

**Figure 13:**
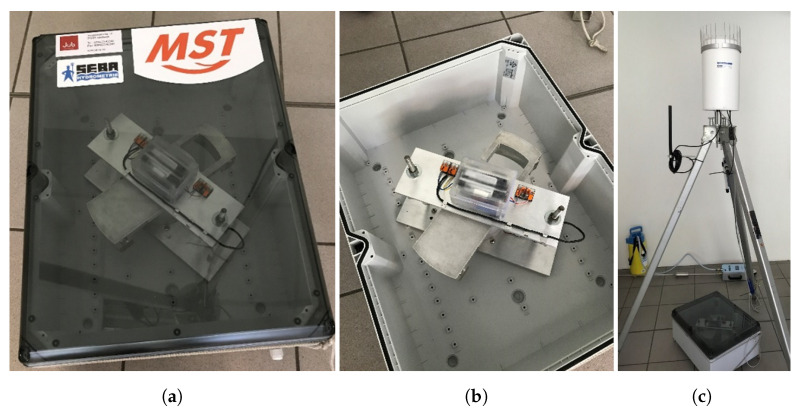
(**a**) Housing of the final pendulum converter system, (**b**) pendulum based converter and energy management and storage circuits integrated in the housing, (**c**) components of the final prototype with precipitation sensor, electronic platform, and temperature sensor.

In addition to laboratory prototyping of the pendulum transducer, charging circuit for the batteries, and hydrometric sensors, field measurements were conducted on a river in Chemnitz, Germany. For this purpose, the system with magnetic pendulum transducers first had to be robustly integrated into a watertight enclosure of dimensions 500 × 280 × 250 mm, as shown in Figure 13a. An illustration of the converter and the energy management and storage circuits are illustrated in Figure 13b. The pendulum converter prototype was finally equipped with a SlimlogCom 4G platform and fitted with the two sensors for precipitation RG50 and water level and temperature sensor DST-22. For the energy supply, the system was also expanded with buffer batteries. The electronic platform is compactly integrated in the housing of the RG50 (see Figure 13c).

**Figure 14:**
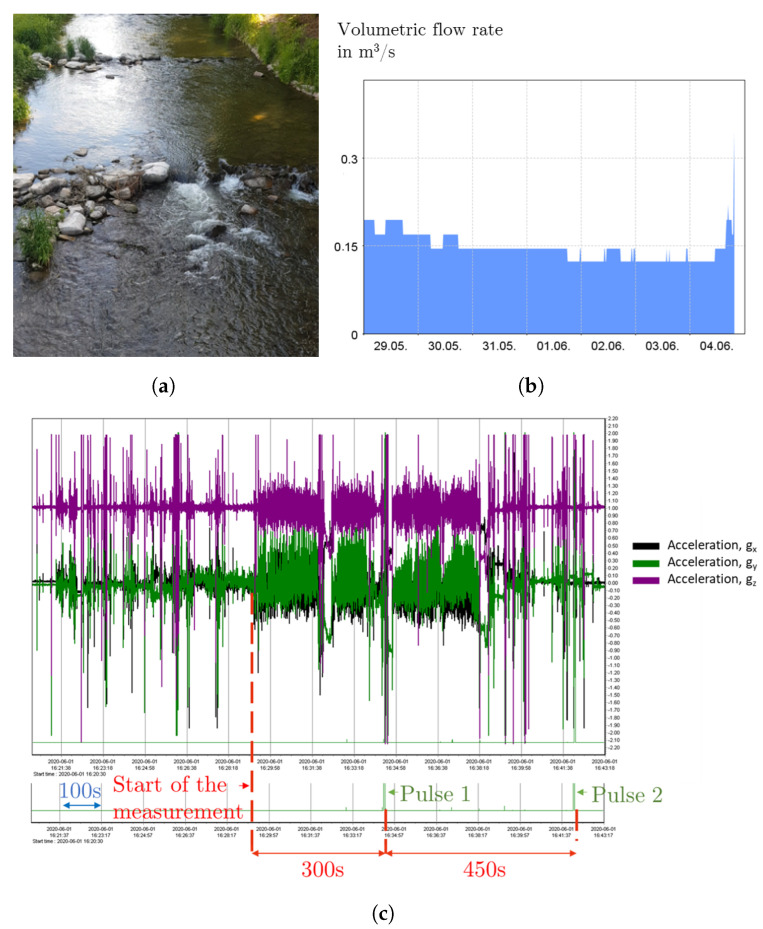
(**a**) Field measurement location, (**b**) volumetric flow rate of the river during the measurement period according to data provided by the Saxon State Office for Environment, Agriculture and Geology, (**c**) acceleration values and output voltage from the MSR 160 data logger.

Figure 14a shows the field measurement position. By considering a kinematic viscosity of approximately 1 mm${}^{2}$/s and *Q* = 0.15 m${}^{3}$/s (see Figure 14b), the Reynolds number of the river is typically $R_{e}$ > 10${}^{6}$.

The housing containing the converter was attached with a cord to a static frame fixed to the selected obstacle in the river. The length of the cord is equal to the determined recirculation zone based on the finite element analysis. Further, a data logger (MSR 160) was attached to the housing, and the acceleration along the three axes and the voltage output from the converter were measured to determine the time between pulses and therefore the harvested energy.

Recorded signals from the data logger are shown in Figure 14c, which include the measured acceleration in the three axes and the output voltage form the kinetic converter. It was found that the pulse repetition rate ranged from approximately 5 to 7 min. It should be noted that theoretically and based on the daily current consumption of the load determined in the previous section, which is approximately 4–4.3 mAh at 7 V, the lifetime of the autonomous sensor system can be determined. Table 6 shows the estimated lifetime of the system as function of the time between pulses that can be generated by the kinetic converter. Theoretically, the system has an infinite lifetime if the generator is able to generate an average of one pulse/10 min or more. It is worth noting that these results are calculated without considering self-discharge of the batteries and for a daily electric charge consumption of the sensor system of 4.2 mAh at 7 V. Further, according to measurements done at different locations, the system’s performance was similar and confirms the reproducibility of the obtained values.

## 5. Discussion

Compared to the state of the art, the proposed conversion principle is characterized by the fact that even low water flow excitations can be converted. A high specific converter power density can be achieved due to the spring energy storage. In addition, the pendulum with magnetic spring allows the converter to operate even under unidirectional excitation. Without the magnetic spring principle, this would not be possible because the pendulum remains in a stationary state. Based on the novel approach, redundant battery-operated storage concepts can be optimized in the future by energy-autonomous conceptions of measuring stations. Likewise, a complete abandonment of redundant storage concepts is conceivable, which would allow for more cost-efficient maintenance intervals. The system represents an interesting solution for seamless monitoring of dangerous environmental phenomena such as extreme flood events with threats to humans, animals, and property or water quality issues (e.g., overturning of water bodies).

## 6. Conclusions

The present paper proposes a novel energy harvesting approach for harvesting energy from water flow to supply hydrometric monitoring systems. The converter consists of a rotating pendulum connected to a bi-directional mechanical system, spiral torsion spring, gears, and a DC generator having a similar working principle to that of kinetic wristwatches. The converter performance was evaluated for different load resistances by a model based approach. For this, a physical model is developed for the whole converter in Matlab Simscape. Taking into account all events and components, the daily consumption of the hydrometric monitoring system was experimentally determined. It was found that the daily current consumption of the electronic platform with RG 50 with a 6 V supply is approximately 4.2 mAh.

In addition, corresponding energy management and storage circuits were designed and fabricated. A prototype of the complete system consisting of the novel converter, energy management and storage circuits, and a data logger platform with a modem (SlimLogCom 4G) and a precipitation sensor RG50 was fabricated. For the characterization of the overall system, laboratory tests were conducted, in addition to testing in a river. Laboratory characterization shows that the converter can harvest approximately 7.2 mAh and 4.3 mAh electric charge per day at 7 V when considering 6 min and 10 min separation times between two successive releases, respectively. In addition, measurements conducted in a river demonstrated that the harvester could generate at least one pulse in five to seven minutes. Thus, it has been demonstrated that the harvester can fully supply the hydrometric monitoring system, thereby avoiding the need for external power sources.

In comparison to previous energy harvesting systems for rivers, the proposed energy harvesting solution could enable the use of hydrometric monitoring systems under various conditions and avoid the need to replace batteries by preserving and utilizing the collected energy. This solution is advantageous in that the harvesting system remains enclosed within a stable housing with no external interface to the flowing water, resulting in a high level of reliability. The system under consideration has the potential to allow the use of hydrometric monitoring systems under a variety of conditions and even with additional sensors as well as a higher number of communication events, while eliminating the need for battery replacement.

## 7. Patents

A part of this work has been patented.

## Figures and Tables

**Table 1:** Parameters used in the physical model.

Component	Parameter	Value
Pendulum	Weight	850 g
	Diameter	25 cm
	Weight of the added mass	490 g
Gears	Transmission ratio spring-generator	0.0211
	Transmission ratio pendulum-spring	1
Spring	Material	X10CrNi18-8
	Spring rate	0–3.5 N·m
	Plate thickness	0.2 mm
	Plate width	10 mm
	Outer diameter	48 mm
	Inner diameter	20 mm
DC generator	Rotor Inertia	1.68 × 10${}^{-7}$ kg·m${}^{2}$
	Armature resistance	13 Ω
	Armature inductance	0.009 H
	Torque constant	0.00963 N·m/A

**Table 2:** Daily current consumption of the UniLogCom with RG50 sensor with a 6 V supply.

Type of Load	Number of Events	Current Consumption per Event in mAh	Total Current Consumption in mAh
FTP push	1	1.28330	1.28330
Logging	2	0.00143	0.00285
Time slot	0.143	4.79140	0.68517
Rain pulses	100.000	0.00069	0.06900
Quiescent current	-	-	2.16000
Daily current consumption	-	-	4.200

**Table 3:** Daily current consumption of the UniLogCom with RG50 sensor with a 12 V supply.

Type of Load	Number of Events	Current Consumption per Event in mAh	Total Current Consumption in mAh
FTP push	1	0.83140	0.83140
Logging	2	0.00085	0.00170
Time slot	0.143	2.20955	0.31597
Rain pulses	100.000	0.00034	0.03400
Quiescent current	-	-	2.04000
Daily current consumption	-	-	3.223

**Table 4:** Components used in the battery charging circuit.

Component	Number	Details
L-ion battery	2	3.7 V 3000 mAh Samsung ICR18650-30B
Voltage protection module	1	Overcharge voltage: 4.25–4.35 V ± 0.05 V Over discharge voltage: 2.3–3.0 V ± 0.05 V Maximum continuous current: 3 A Maximum peak current: 5 A
Constant-current/ constant-voltage battery charger	1	Based on LT1512 Output voltage: 8.4 V Minimum input voltage: 2 V

**Table 5:** Characterization of the converter voltage output under different load resistances.

Load Resistance	Required Turns of the Pendulum before Release	Harvested Power per Pulse at 8.4 V in mAh	Harvested Power per Pendulum Turn at 8.4 V in mAh
20 Ω	not working	0	0
43 Ω	working continuously	0.0012	0
82 Ω	1	0.0058	0.0058
560 Ω	9	0.0228	0.0025
820 Ω	9	0.0206	0.0023
1.2 kΩ	11	0.0287	0.0026
1.5 kΩ	11	0.0241	0.0021
2.7 kΩ	11	0.0294	0.0026
4.7 kΩ	11	0.0303	0.0027
8.2 kΩ	11	0.0250	0.0022
12 kΩ	11	0.0249	0.0022
22 kΩ	11	0.0265	0.0024

**Table 6:** Estimated system lifetime as function of the pulse frequency.

Time between Pulses in Minutes	Harvested Electric Charge at 7 V in mAh in Year	Estimated System Lifetime in Year
5	3153	unlimited
6	2628	unlimited
7	2253	unlimited
8	1971	unlimited
9	1752	unlimited
10	1576	unlimited
20	788	6.5
30	525	4.7
0	0	3.14

## Nomenclature

Symbol/Abbreviation	Description/Meaning
α	Roll angle [°]
β	Pitch angle [°]
ϕ	Yaw angle [°]
$R_{e}$	Reynolds number
*Q*	Volume flow rate [m${}^{3}$/s]
*l*	Length of the river [m]
μ	Kinematic viscosity [m${}^{2}$/s]
*k*	Turbulent kinetic energy [J/kg] = [m${}^{2}$s${}^{-2}$]
ϵ	Rate of dissipation of the turbulent kinetic energy [m${}^{2}$/s${}^{-3}$]
CAD	Computer-Aided Design
CFD	Computational Fluid Dynamics
DC	Direct Current
DPEH	Downstream Piezoelectric Energy Harvester
FTP	File Transfer Protocol
MFC	Micro Fiber Composite
PEH	Piezoelectric Energy Harvester
PVC	Polyvinyl Chloride
RANS	Reynolds-Averaged Navier–Stokes
RG50	Precipitation Sensor
SEPIC	Single-Ended Primary Inductance Converter
UPEH	Upstream Piezoelectric Energy Harvester

## References

[1] Rahimzadeh M., Samadi H., Mohammadi N. (2021). Analysis of Energy Harvesting Enhancement in Piezoelectric Unimorph Cantilevers. Sensors.

[2] Kargar S., Hao G. (2022). An Atlas of Piezoelectric Energy Harvesters in Oceanic Applications. Sensors.

[3] Bradai S., Naifar S., Kanoun O. (2019). Development of a hybrid vibration converter for real vibration source/Entwicklung eines Hybrid-Vibrationswandlers für eine echte Schwingungsquelle. Tech. Mess..

[4] Bradai S., Naifar S., Trigona C., Baglio S., Kanoun O. (2021). An electromagnetic/magnetoelectric transducer based on nonlinear RMSHI circuit for energy harvesting and sensing. Measurement.

[5] (1997). Communication from the Commission and Communication from the Commission. Energy for the Future: Renewable Sources of Energy. White Paper for a Community Strategy and Action Plan.

[6] Pop-Vadean A., Pop P., Latinovic T., Barz C., Lung C. (2017). Harvesting energy an sustainable power source, replace batteries for powering WSN and devices on the IoT. IOP Conf. Ser. Mater. Sci. Eng..

[7] Satharasinghe A., Hughes-Riley T., Dias T. (2020). A Review of Solar Energy Harvesting Electronic Textiles. Sensors.

[8] Kishore R., Priya S. (2018). A Review on Low-Grade Thermal Energy Harvesting: Materials, Methods and Devices. Materials.

[9] Naifar S., Bradai S., Viehweger C., Kanoun O. (2017). Survey of electromagnetic and magnetoelectric vibration energy harvesters for low frequency excitation. Measurement.

[10] Song R., Hou C., Yang C., Yang X., Guo Q., Shan X. (2021). Modeling, Validation, and Performance of Two Tandem Cylinder Piezoelectric Energy Harvesters in Water Flow. Micromachines.

[11] Kong H., Roussinova V., Stoilov V. (2018). Renewable energy harvesting from water flow. Int. J. Environ. Stud..

[12] Shaikh F., Zeadally S. (2016). Energy harvesting in wireless sensor networks: A comprehensive review. Renew. Sustain. Energy Rev..

[13] Raja Singh R., Raj Chelliah T., Agarwal P. (2014). Power electronics in hydro electric energy systems—A review. Renew. Sustain. Energy Rev..

[14] Ferrarese G., Malavasi S., Williams R. (2015). Energy Harvesting: Technology, Methods and Applications.

[15] Azevedo J., Lopes J. (2016). Energy harvesting from hydroelectric systems for remote sensors. AIMS Energy.

[16] Morais R., Matos S., Fernandes M., Valente A., Soares S., Ferreira P., Reis M. (2008). Sun, wind and water flow as energy supply for small stationary data acquisition platforms. Comput. Electron. Agric..

[17] Taylor G., Burns J., Kammann S., Powers W., Welsh T. (2001). The Energy Harvesting Eel: A small subsurface ocean/river power generator. IEEE J. Ocean. Eng..

[18] Kamenar E., Zelenika S., Blazevic D., Samanic I. River flow energy harvesting by employing piezoelectric eels. Proceedings of the 14th EUSPEN International Conference.

[19] Shan X., Song R., Liu B., Xie T. (2015). Novel energy harvesting: A macro fiber composite piezoelectric energy harvester in the water vortex. Ceram. Int..

[20] Song R., Shan X., Lv F., Xie T. (2015). A study of vortex-induced energy harvesting from water using PZT piezoelectric cantilever with cylindrical extension. Ceram. Int..

[21] HY2120 Data Sheet 2-Cell Lithium-ion/ Polymer Battery Packs Protection ICs. https://www.hycontek.com/wp-content/uploads/DS-HY2120_EN.pdf.

